# *Elioraea tepida,* sp. nov., a Moderately Thermophilic Aerobic Anoxygenic Phototrophic Bacterium Isolated from the Mat Community of an Alkaline Siliceous Hot Spring in Yellowstone National Park, WY, USA

**DOI:** 10.3390/microorganisms10010080

**Published:** 2021-12-31

**Authors:** Mohit Kumar Saini, Shohei Yoshida, Aswathy Sebastian, Eri Hara, Hideyuki Tamaki, Nathan T. Soulier, Istvan Albert, Satoshi Hanada, Marcus Tank, Donald A. Bryant

**Affiliations:** 1 Department of Biological Sciences, Tokyo Metropolitan University, Tokyo 192-0397, Japan; mohitvtm@gmail.com (M.K.S.); rhcp.mariabeetle@gmail.com (S.Y.); shanada2@icloud.com (S.H.); 2 The Huck Institutes for the Life Sciences, The Pennsylvania State University, University Park, PA 16802, USA; azs13@psu.edu (A.S.); iua1@psu.edu (I.A.); 3 Bioproduction Research Institute—National Institute of Advanced Industrial Science and Technology (AIST), 1-1-1, Higashi, Tsukuba 305-8566, Japan; hara-eri@aist.go.jp (E.H.); tamaki-hideyuki@aist.go.jp (H.T.); 4 Department of Biochemistry and Molecular Biology, The Pennsylvania State University, University Park, PA 16802, USA; riftreiluos@gmail.com; 5 DSMZ-German Culture Collection of Microorganisms and Cell Cultures, GmbH Inhoffenstraße 7B, 38124 Braunschweig, Germany

**Keywords:** aerobic anoxygenic phototrophic bacteria, chlorophototroph, thermophile, hot spring, bacteriochlorophyll, *Alphaproteobacteria*

## Abstract

Strain MS-P2^T^ was isolated from microbial mats associated with Mushroom Spring, an alkaline siliceous hot spring in Yellowstone National Park, WY, USA. The isolate grows chemoheterotrophically by oxygen-dependent respiration, and light stimulates photoheterotrophic growth under strictly oxic conditions. Strain MS-P2^T^ synthesizes bacteriochlorophyll *a* and the carotenoid spirilloxanthin. However, photoautotrophic growth did not occur under oxic or anoxic conditions, suggesting that this strain should be classified as an aerobic anoxygenic phototrophic bacterium. Strain MS-P2^T^ cells are motile, curved rods about 0.5 to 1.0 μm wide and 1.0 to 1.5 μm long. The optimum growth temperature is 45–50 °C, and the optimum pH for growth is circum-neutral (pH 7.0–7.5). Sequence analysis of the 16S rRNA gene revealed that strain MS-P2^T^ is closely related to *Elioraea* species, members of the class *Alphaproteobacteria*, with a sequence identity of 96.58 to 98%. The genome of strain MS-P2^T^ is a single circular DNA molecule of 3,367,643 bp with a mol% guanine-plus-cytosine content of 70.6%. Based on phylogenetic, physiological, biochemical, and genomic characteristics, we propose this bacteriochlorophyll *a*-containing isolate is a new species belonging to the genus *Elioraea*, with the suggested name *Elioraea* *tepida*. The type-strain is strain MS-P2^T^ (= JCM33060^T^ = ATCC TSD-174^T^).

## 1. Introduction

Aerobic anoxygenic phototrophic bacteria (AAPB) are bacteria that grow chemoheterotrophically by oxygen-dependent respiration, but they also synthesize photosynthetic reaction centers and light-harvesting complexes containing bacteriochlorophyll (BChl) *a* under oxic conditions [1,2]. Although AAPB can produce a photosynthetic apparatus, AAPB are unable to reduce carbon dioxide; light usually stimulates growth slightly under chemoorganoheterotropic conditions. Many species of AAPB have been found in a variety of habitats, including oceans, freshwater lakes and rivers, and soils [3]. Although most AAPB are mesophiles, some species are moderate thermophiles isolated from hot springs. Examples include *Porphyrobacter tepidarius* [4], *Porphyrobacter cryptus* [5], and *Rubritepida flocculans* [6].

AAPB do not form a monophyletic clade but are widely distributed within the classes *Alphaproteobacteria*, *Betaproteobacteria,* and *Gammaproteobacteria* [7]. Phylogenetically, AAPB are often more closely related to non-phototrophic bacteria than to phototrophic bacteria [3]. The family *Acetobacteraceae* in the *Alphaproteobacteria* contains several examples of AAPB, e.g., *Roseococcus thiosulfatophilus* [8], *Humitalea rosea* [9], *Craurococcus roseus* [10], *Roseomonas aestuarii* [11], and *Acidiphilium rubrum* [12]; these AAPBs are interspersed among non-phototrophic bacteria. Of the AAPB belonging to the *Acetobacteraceae*, *Rubritepida flocculans* was once thought to be the sole thermophilic species [6]. Interestingly, *Rubritepida flocculans* cells grown at high temperatures do not produce BChl *a*, but BChl *a* and carotenoids are produced when cells are grown at 30 °C.

The type species of the genus *Elioraea,* i.e., *Elioraea tepidiphila*, is a slightly thermophilic bacterium that was isolated from a hot spring in the Furnas area of the island of São Miguel in the Azores [13]. The genus *Elioraea* belongs to the class *Alphaproteobacteria,* which was proposed to be the type genus of a separate family, *Elioraeaceae,* which was suggested to be a closely related sister clade to *Acetobacteraceae* in the order *Rhodospirillales* [13,14]. However, more recently the genus *Elioraea* was moved back into the family *Acetobacteraceae* [15,16]. The optimal growth temperature range for *E. tepidiphila* is 45 to 50 °C and the optimal pH is between pH 8.0 and 8.5. Initially, bacteriochlorophyll (BChl) *a* and carotenoids were not detected [13,14]. However, subsequent sequencing of the *E. tepidi-phila* genome (GenBank Project Accession NZ_ARKI00000000) revealed these capabilities, which were then verified experimentally by others (see Habib et al. [14]).

Previous studies reported the occurrence of an organism phylogenetically related to *E. tepidiphila* in microbial mats associated with Mushroom Spring in Yellowstone National Park (YNP), WY, USA [17,18]. Spectroscopic measurements revealed that this new isolate, strain MS-P2^T^, produces substantial amounts of BChl *a* even under aerobic conditions in the light at high temperature [18], which suggested that this new isolate should be considered to be a thermophilic member of the AAPB. Based on its growth at elevated temperatures and its relationship to *E. tepidiphila*, this new organism was named “*Candidatus* Elioraea thermophila” strain MS-P2^T^ by Tank et al. [18].

More recently still, another organism closely related to *E. tepidiphila* was isolated from a hot spring in Yunnan province, China [14]. These authors were apparently unaware of the previous use of the species epithet “thermophila” to describe the organism from Mushroom Spring, and they also named their isolate *E. thermophila*. The authors reported that both *E. thermophila* and *E. tepidiphila* could synthesize BChl *a* and carotenoids and that both possessed *pufLM* genes. Importantly, Habib et al. [14] also reported that their *E. thermophila* isolate could grow photoautotrophically when thiosulfate or hydrogen served as electron donors. This is possibly because this bacterium is thus far the only *Elioraea* species that has genes encoding phosphoribulokinase and a type-1 ribulose 1,5-bisphosphate carboxylase-oxygenase (RuBisCO), and thus it should have the capacity to fix CO_2_ by the Calvin–Benson–Bassham cycle. 

A mesophilic *Elioraea* species that promotes plant growth, *E. rosea*, was also recently described [19]. This bacterium, which was isolated from the floodwater of a paddy field in South Korea, is strictly aerobic, motile by swimming, and pink-pigmented with a growth temperature optimum of 28 °C. Although it has not yet been validly described, yet another thermophilic *Elioraea* sp., strain “Yellowstone”, was isolated from the runoff channel of Octopus Spring in YNP at 50 °C, and an incomplete, draft genome sequence is available [20].

In this manuscript, we describe the major characteristics of the AAPB isolate MS-P2^T^ from Mushroom Spring and compare its properties with those of *E. tepidiphila*, *E. thermophila*, *E. rosea*, and *Elioraea* sp. “Yellowstone”. Additionally, phylogenomic analyses are presented that are based upon the complete genomic sequence data for the Mushroom Spring isolate. Based on its phenotypic and phylogenetic characteristics, we propose that this isolate represents a new species within the genus *Elioraea* with the suggested name, *Elioraea tepida*.

## 2. Materials and Methods

### 2.1. Strain Isolation and Cultivation Conditions

‘*Chloracidobacterium thermophilum* Midnight medium’ (CTM medium) [21,22] supplemented with 0.8% (*w*/*v*) agar (pH 7.0) was used as the basal medium for the isolation. A microbial mat sample collected from Mushroom Spring in the Lower Geyser Basin of Yellowstone National Park, WY, USA (GPS coordinates: Lat.: 44.5387, Long.: –110.798) was inoculated into sterile liquid CTM medium, and the culture was incubated in a beaker covered with aluminum-foil under continuous illumination from an incandescent light (approx. 20–50 µmol photons m^−2^ s^−1^) at 50 °C for >10 days. Diluted samples from the enrichment culture were mixed with liquified agar prepared with CTM medium at 45 °C (tempered agar), and the agar mixtures were then poured into Petri dishes. After cooling to solidify the agar, the plates were incubated at 50 °C. Pink colonies that formed in the solidified agar medium within a week were picked up with a sterilized Pasteur pipet, resuspended into 1.0 mL of sterile distilled water, and mixed with tempered agar in CTM medium, cooled to solidify the agar, and incubated at 50 °C. This procedure was repeated until axenic cultures were obtained. Liquid cultivation was also successful in basal CTM medium containing succinate, acetate, and yeast extract (1.0 g L^–1^ of each). The axenic cultures could be maintained as 60-mL cultures in 100-mL Erlenmeyer flasks covered with aluminum foil, with or without shaking (150 rpm) at 50 °C (see Supplementary Figure S1A).

### 2.2. Microscopy and Analytical Procedures

The size and shape of the cells of strain MS-P2^T^ were determined by phase-contrast microscopy. Autofluorescence from BChl *a* of cells of strain MS-P2^T^ was visualized under an epifluorescence microscope (Nikon Eclipse E600 (NIS-Elements software D), Nikon Xenon power supply XPS-100, monochromatic CMOS camera (Orcaflash 4.0, Hamamatsu), Filterset Excitation: 350–510 nm dichroic mirror:665 nm/Emission: 830 nm LP (SEMROCK, Rochester, NY, USA)). 

Absorbance spectra were measured with a Shimadzu model UV-1800 spectrophotometer (Shimadzu Corporation, Kyoto, Japan). Pigments were extracted with acetone–methanol (7:2, *v*/*v*), and the absorbance spectra of extracts were measured to assess in a preliminary manner the pigmentation of cells. Extracted pigments were also analyzed by reversed-phase, high-performance liquid chromatography (HPLC) as described [23]. Pigments were extracted with acetone–methanol (7:2, *v*/*v*) and were filtered with a 0.2-µm polytetrafluoroethylene, single-use filter device (Cytiva, Marlborough, MA, USA) prior to injection into the column (25 cm × 4.6 mm MilliporeSigma™ Supelco™ Discovery™ 5-µm C-18 column; Fisher Scientific, Hampton, NH, USA). The filtered pigment solution was analyzed with an Agilent 1100 HPLC system equipped with a diode-array detector (Santa Clara, CA, USA), as previously described [24]. The identities of pigments were determined by comparison of elution times and in-line absorbance spectra to those of authenticated standards that are maintained in the laboratory. 

Gram-staining was performed by using the Favor-G kit (Nissui Pharmaceutical, Tokyo, Japan). Production of catalase was assessed by measuring bubble production in 3% (*v*/*v*) H_2_O_2_, and cytochrome *c* oxidase activity was assessed by using the oxidase reagent (bioMérieux, Marcy l’Etoile, France). The presence of other selected enzymes was tested using the APIZYM system (bioMérieux, Marcy l’Etoile, France). The ability to oxidize a variety of substrates was tested using the Biolog GEN III plate system (Biolog, Hayward, CA, USA). The Biolog Gen III system tests 71 carbon substrates and 23 potentially inhibitory growth conditions in a 96-well microtiter plate format. Cells were resuspended at a recommended concentration in a proprietary “inoculation solution”, that was amended to contain 0.1 g/L yeast extract. The cell suspension was then pipetted into each of the 96 wells, which included positive and negative control wells. Each well contained a carbon substrate and other nutrients and buffer or contained a potential growth-inhibiting condition (e.g., salt, pH, antibiotic, etc.). The plates were incubated at 45 °C for 24 to 48 h, and the OD_590_ of each well was recorded at 8-h intervals. Positive wells turned purple in color due to the reduction of a tetrazolium redox dye, indicating growth and/or oxidation of the included substrate. The small amount of yeast extract added to the inoculation medium was insufficient to produce a positive color reaction. 

Finally, fatty acid methyl esters, respiratory quinones, and the GC content of the DNA were analyzed according to previously described procedures [25]. Polar lipids were extracted using the Bligh–Dyer method and were analyzed by two-dimensional thin-layer chromatography as previously described [26,27]. 

### 2.3. Genome Sequencing and Bioinformatic Analyses

Total genomic DNA of strain MS-P2^T^ was extracted, sequenced, and assembled as previously described [28]. The genome of strain MS-P2^T^ was sequenced using the PacBio Sequel platform. Sequence assembly was performed using a Canu 1.8 assembler, yielding a high-quality, closed circular genome. The completeness and contamination of the genome were checked using the online version of CheckM implemented in the Kbase software and data platform [29]. The assembled genome was annotated by the RAST annotation system (Rapid Annotation using SEED Technology) [30,31,32] as well as by using the NCBI Prokaryotic Genome Annotation Pipeline (www.ncbi.nlm.nih.gov/genome/annotation_prok/ (accessed on 15 July 2021)) [33,34,35] to predict the number of coding genes, total RNA genes, and functional genes. The gene locus designations and gene names used in the manuscript are those from the NCBI Prokaryotic Genome Annotation Pipeline. Average nucleotide identity (ANI) values were calculated by the OrthoANI algorithm method as implemented in the OAT software package [36]. Digital DNA–DNA hybridization (dDDH) values were determined with the in-silico genome-to-genome distance calculator (GGDC2.1; http://ggdc.dsmz.de/distcalc2.php (accessed on 26 May 2021)) using the alignment method blast+ [37,38,39]. Genomes of type species belonging to the genus *Elioraea* (*E. tepidiphila* TU-7^T^ [13], *E. thermophila* YIM 72297^T^ [14], and *E. rosea* PF-30^T^ [19]) were compared with the genome of strain MS-P2^T^. The MS-P2^T^ genome was also compared to the genome of *Elioraea* sp. strain “Yellowstone” [20]. 

A phylogeny based on concatenated proteins was constructed using a set of 49 universally conserved proteins/gene as defined by COG (Clusters of Orthologous Groups) gene families on the Kbase platform using the “SpeciesTree builder version 2.2.0” [40]. This analysis included the genomes of five *Elioraea* spp. with a set of closely related genomes selected from the public KBase genomes import from RefSeq. Relatedness was determined by alignment similarity to a select subset of 49 COG domains. FastTree2 was used to prepare the phylogenetic tree [40]. A list of the 49 proteins used, as well as other information, can be found online at https://narrative.kbase.us/#catalog/apps/SpeciesTreeBuilder/insert_set_of_genomes_into_species_tree/release (accessed on 17 December 2021).

## 3. Results and Discussion

### 3.1. Isolation and Initial Cultivation

Strain MS-P2^T^ was isolated from the microbial mats associated with the main runoff channel at Mushroom Spring in the Lower Geyser Basin of Yellowstone National Park, WY, USA (GPS coordinates: Lat.: 44.5387, Long.: −110.798). The temperature at the sampling site was 52 °C, and the pH was ~8.0. In this hot spring, the microbial mats mainly consist of thermophilic cyanobacteria of the genus *Synechococcus* [41,42,43] and phototrophic *Chloroflexota* from the genera *Roseiflexus*, *Chloroflexus*, “*Candidatus* Roseilinea”, and “*Candidatus* Chloranaerofilum” [17,18,42,43,44]. Several other chlorophototrophic organisms, including two *Chloracidobacterium* species, *C. thermophilum* and *C. aggregatum* [21,22,23], “*Candidatus* Thermochlorobacter aerophilum” [45], and at least four chlorophototrophic members of the *Proteobacteria* also occur in these mats [18]. Strain MS-P2^T^ was isolated as described in Section 2.1 of the Materials and Methods using CTM medium solidified with 0.8% (*w*/*v*) agar at pH 7.0. Pink colonies appeared within a week and were purified by restreaking. Liquid cultures were pinkish-orange in color, and concentrated cells were bright pink (Supplementary Figure S1A,B).

### 3.2. Phenotypic, Biochemical, and Chemotaxonomic Characterization

**Figure 1:**
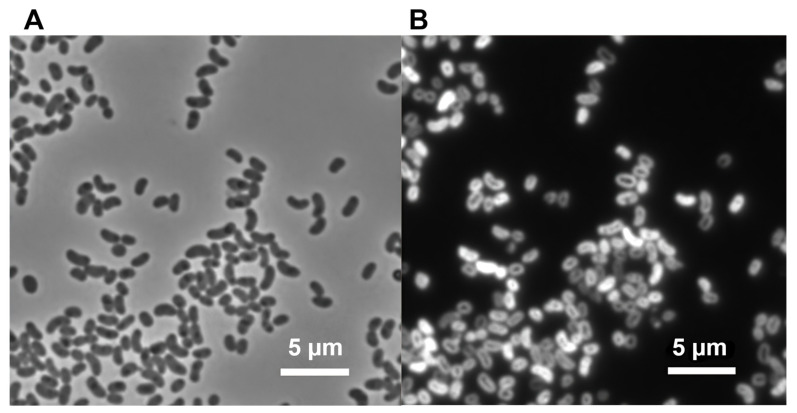
Phase-contrast fluorescence and microscopic images of *E. tepida* strain MS-P2^T^. (**A**) Phase-contrast micrograph of strain MS-P2^T^ showing curved, vibrio-shaped cells that multiply by binary fission. (**B**) Fluorescence micrograph of the same field as panel (**A**). Scale bars = 5 μm.

Cells of strain MS-P2^T^ grown in liquid CTM medium were motile, curved rods with dimensions of 0.5 to 1.0 μm (width) by 1.0 to 1.5 μm (length) (Table 1). Strain MS-P2^T^ cells stained Gram-negative and were positive for both catalase and cytochrome *c* oxidase. Division occurred by binary fission (Figure 1A,B). When viewed by epifluorescence microscopy with a filter set specific for BChl *a*, cells exhibited intense autofluorescence, which suggested that the cells contain a substantial amount of BChl *a* (Figure 1B).

**Figure 2:**
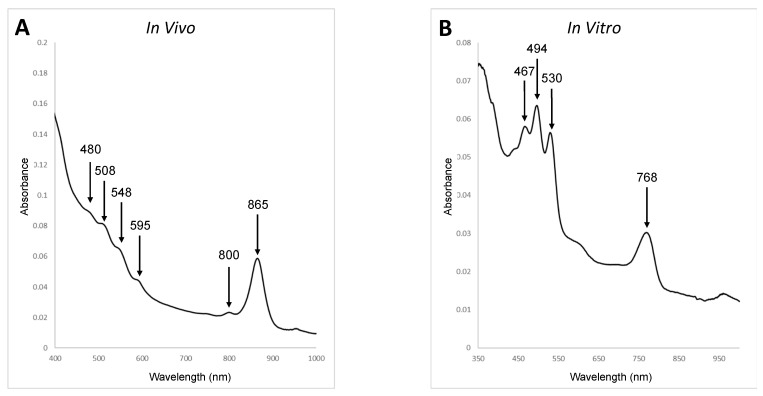
Absorbance spectra of *E. tepida* strain MS-P2^T^. (**A**). An in vivo absorbance spectrum of strain MS-P2^T^. The cells were grown in CTM medium under aerobic conditions in the dark and then were disrupted by ultrasonication. Absorbance maxima are indicated by the arrows. (**B**). Absorbance spectrum of pigments extracted from cells with acetone–methanol (7:2, *v*/*v*). The absorbance maxima indicate the presence of BChl *a* and carotenoids. For additional details, see text. Similar results were obtained with cells grown in the light.

The in vivo absorbance spectrum of strain MS-P2^T^ cells that had been disrupted by ultrasonication in phosphate-buffered saline buffer (0.137 M NaCl, 0.0081 M Na_2_HPO_4_, 0.00268 M KCl, 0.00147 M KH_2_PO_4_ pH 7.0) is shown in Figure 2. The in vivo spectrum had distinctive absorbance peaks at 800 and 865 nm in the infrared region and a smaller maximum at 590 nm in the visible region, consistent with the presence of BChl *a* (Figure 2). The spectrum also suggested that strain MS-P2^T^ has light-harvesting complex 1 (LH1) but that it might lack light-harvesting complex 2 (LH2; however, see below). The three absorbance maxima between 450 and 550 nm suggest that the isolate also produces carotenoids. Pigments were extracted with acetone-methanol (7:2, *v*/*v*) and an absorbance spectrum was also recorded. The presence of BChl *a* (Q_y_ band absorbance maximum at 770 nm) and carotenoids (absorbance maxima at 467, 494, and 530 nm) (Figure 2) were also detected in the spectrum of the pigment extract. To confirm this preliminary assessment, extracted pigments were analyzed by reversed-phase HPLC as described [24]. BChl *a* esterified with phytol, a small amount of bacteriopheophytin *a,* and the carotenoids spirilloxanthin (major) and 3,4-dehydrorhodopin (minor) were identified by comparison to authentic standards (Supplementary Figure S2).

Strain MS-P2^T^ was able to grow chemoheterotrophically under aerobic conditions in the dark but was unable to grow photoautotrophically under oxic or anoxic conditions in the light. Cells grown in light grew slightly faster than cells grown under the same aerobic chemoheterotrophic conditions in the dark. 

**Figure 3:**
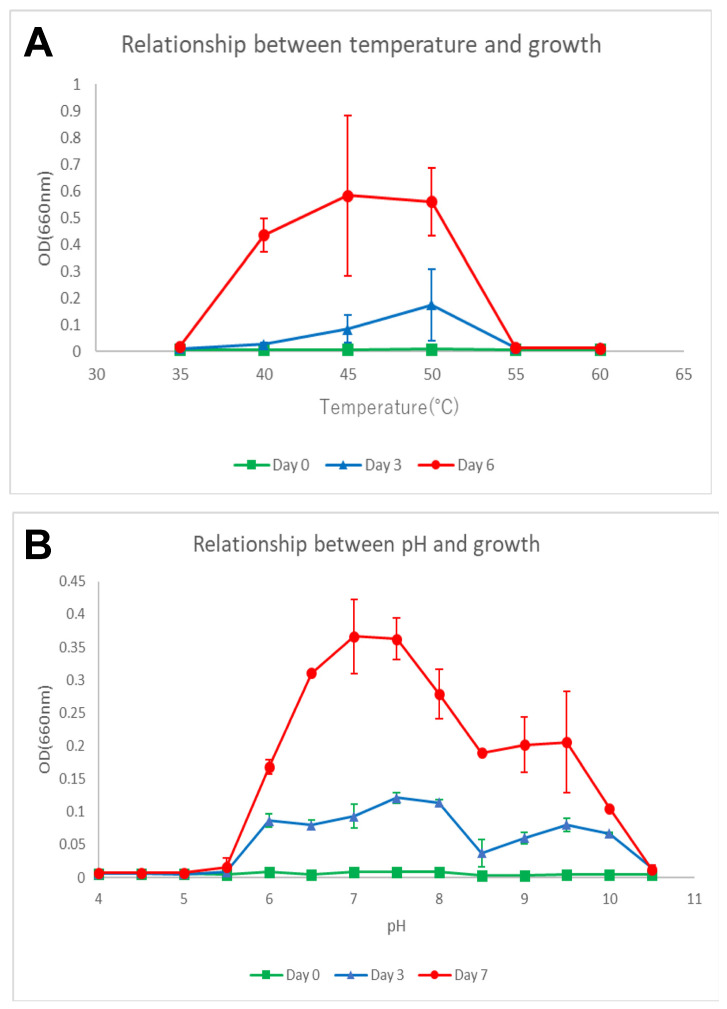
Growth behavior of *Elioraea tepida* strain MS-P2^T^ as a function of temperature (**A**) and pH (**B**). Growth of this strain occurred at temperatures greater than 35 °C and lower than 55 °C with an optimum between 45 and 50 °C. Growth occurred over a wide range of pH values above pH 5.5 and below pH 10.5, with an optimum value at pH 7.0 to 7.5. The OD_660_ values at time zero (green line), after three days of growth (blue line), and 6 (temperature) or 7 days (pH) of growth (red line) are plotted. The plotted values are the mean and standard deviation values from triplicate cultures.

The growth temperature range of strain MS-P2^T^ was examined by measuring growth at 35, 45, 50, 55, and 60 °C by measuring the OD_660_ of cultures incubated in 60 mL of liquid CTM medium containing succinate, acetate, and yeast extract (contained 1.0 g L^−1^, respectively) in 100-mL aluminum-foil-capped flasks, with shaking at 150 rpm. The optimal growth of strain MS-P2^T^ occurred between 45 and 50 °C (Figure 3A). The strain also grew at 40 °C but did not grow above 55 °C or below 35 °C. Thus, the growth temperature range can be described as ~40 °C and higher but less than 55 °C (Table 1).

The pH range for growth was tested from pH 4.0 to 10.5 at 50 °C in the CTM medium by using appropriate buffering agents: 10 mM 2-(N-morpholino)ethanesulfonic acid (MES), 10 mM (4-(2-hydroxyethyl)-1-piperazineethanesulfonic acid (HEPES), 10 mM Bicine, and 50 mM N-cyclohexyl-3-aminopropanesulfonic acid (CAPS). Strain MS-P2^T^ grew at pH values between 6.0 and 10.0 but was unable to grow at pH 5.5 or at pH 10.5. The optimum pH for growth was pH 7.0 to 7.5 (Figure 3B; Table 1).

Succinate and acetate (1.0 g L^−1^) clearly promoted growth of strain MS-P2^T^ when added to CTM media containing yeast extract (1.0 g L^−1^). Based on results from Biolog GN2 and GEN3 testing, strain MS-P2^T^ was able to oxidize a wide variety of compounds, including 3-methyl-d-glucose, citric acid, d-arabitol, d-fructose, d-fucose, d-galactose, d-mannose, d-melibiose, d-psicose, d-sorbitol, glucuronamide, hydroxybutyric acid, l-arabinose, l-proline, methyl pyruvate, *p*-hydroxy-phenylacetic acid, Tween 40, α-d-glucose, α-d-glucose-1-phosphate, β-hydroxy-d, l-butyric acid, and β-methyl-d-glucoside. Gelatin, aesculin, and pectin were hydrolyzed. Anaerobic growth with nitrate (0.1% (*w*/*v*) KNO_3_) as an electron acceptor was not observed. 

Testing with the APIZYM system revealed that alkaline phosphatase, esterase (C4), esterase lipase (C8), leucine arylamidase, valine arylamidase, acid phosphatase, and naphthol-AS-BI-phosphohydrolase were produced.

Strain MS-P2^T^ contains the following fatty acids: 15:0 *iso* (7.8%), 16:0 (12.3%), 18:0 (30.0%), 18:1 *ω*7c (9.9%), 19:0 cyclo *ω*8c (13.0%), 18:1 *ω*7c 11-methyl (22.0%), and 18:0 3-OH (4.7%) (Table 2). The major respiratory quinone was ubiquinone-10. The DNA base composition of the isolate as determined by HPLC was 69.1 mol % ± 0.35 mol % G + C; this is in reasonably good agreement with the actual value of 70.6% calculated from the genome sequence (see below). 

Cells in late-exponential growth phase were harvested for polar lipid analysis. Four major and four minor polar lipids were found in strain MS-P2^T^. Phosphatidylcholine (PC), an unidentified aminophospholipid (AP; possibly phosphatidylethanolamine), and two unidentified aminolipids (AL2 and AL3) comprise the major polar lipids. Diphosphatidylglycerol (DPG), phosphatidylglycerol (PG), an unidentified aminolipid (AL1), and an unidentified phospholipid (PL) were the minor polar lipids (Supplementary Figure S3). The polar lipid composition of strain MS-P2^T^ is similar to the polar lipid composition of *E. tepidiphila* TU-7^T^ [13] and *E. thermophila* YIM 72297^T^ [14].

### 3.3. Genomic Features

The assembled genome of MS-P2^T^ comprises a single circular contig with a total length of 3,367,643 bp exhibiting 100% completeness with no contamination. No plasmids are present. The mol % G + C content calculated from the genome sequence is 70.6%. The genome encoded 3083 protein-coding sequences (CDS), a single rRNA operon, and 46 tRNAs (Table 3). The genome contains a complete set of genes for the synthesis of bacteriochlorophyll *a*, carotenoids of the spirilloxanthin series, and the photosynthetic apparatus (e.g., *pufABLM*, *puhA*). The presence of *acsF* and *bchE* allows BChl synthesis to occur under both oxic and anoxic conditions, respectively [46]. Surprisingly, although the absorbance spectrum of cells suggests the absence of LH2 antenna complexes, the genome encods a *pucBAC* operon that could potentially produce such LH2 complexes. Three terminal oxidases (cytochrome *aa*_3_, *bb*_3_, and *bd* oxidases) are encoded in the genome. Genes (*soxBCDYZ*) for oxidation of thiosulfate are present, but consistent with the absence of photoautotrophic growth, genes for phosphoribulokinase, and ribulose-1,5-bisphosphate carboxylase-oxygenase (RuBisCO), and other enzymes for CO_2_ fixation are absent. Genes encoding nitrogenase and nitrate and nitrite reductase are missing, but genes for urease (*ureABC*) and its assembly (*ureDEFGJ*) are present (Table 3). Finally, *E. tepida* lacks genes for the synthesis of vitamin B_12_ and methionine synthase, but the genome encodes a methionine transporter.

**Figure 4:**
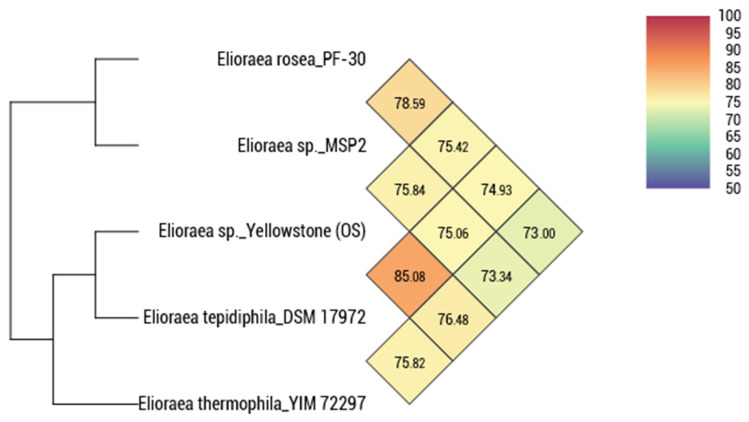
Heat-map and cladogram showing the average nucleotide identity (ANI) values for pairwise comparisons of the genome sequences of five *Elioraea* spp. strains. *Elioraea* sp. strain MS-P2^T^ is very slightly more closely related to *E. rosea* (78.59%) than to the other strains. Otherwise, all pairs are roughly equally dissimilar except for *E. tepidiphila* and *Elioraea* sp. “Yellowstone”. However, the values for all comparisons are well below the threshold of 95–96 ANI expected for members of the same species.

Figure 4 shows the pairwise relationships based on the calculated average nucleotide identity (ANI) for all five *Elioraea* spp. strains for which genome sequence information was available. These data show that *E.* *tepidiphila* and *E. thermophila*, with a pairwise ANI value of 85.08%, are slightly more similar than any other pair of strains, but that all strains are otherwise similarly and distantly related with pairwise ANI values ranging from 73 to 78%. Because ANI values above 95–96% are expected when two strains belong to the same species, strain MS-P2^T^ belongs to the genus *Elioraea* but is obviously different from the other three type-species of this genus. Strain MS-P2^T^ is also distinct from another recently sequenced isolate, *Elioraea* sp. strain “Yellowstone”, from Octopus Spring [20]. Genomic relatedness by digital DNA-DNA hybridization (dDDH) indicates that genomic similarities between strain MS-P2^T^ and *E. rosea* PF-30^T^, *Elioraea* sp. strain “Yellowstone”, *E. tepidiphila* TU-7^T^, and *E. thermophila* YIM 72297^T^ were only 21.90%, 20.50%, 19.80%, and 18.70%, respectively (Table 3). The low pairwise dDDH values (19 to 22%) are far below the threshold values for species-level relatedness (70% dDDH) [47], and thus these genome-wide comparisons strongly support the proposal that strain MS-P2^T^ represents a new species within the genus *Elioraea*. 

**Figure 5:**
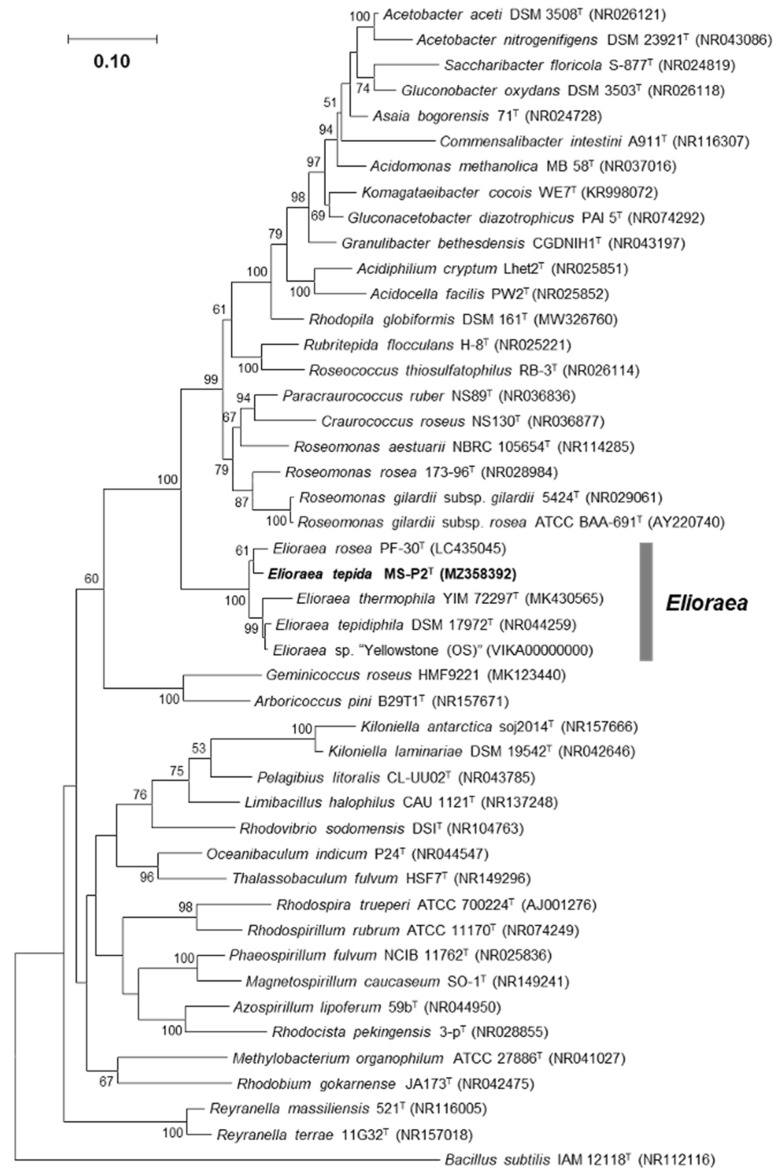
Phylogenetic tree based on 16S rRNA gene sequences constructed by the maximum-likelihood method based on the Kimura 2-parameter model [50] showing the phylogenetic position of strain MS-P2^T^ within the order *Rhodospirillales*. Robustness of the maximum-likelihood tree was tested by bootstrapping (100 resamplings, values > 50 are given at the nodes). *Bacillus subtilis* IAM 12118^T^ was used as an outgroup. The scale bar represents 0.10 substitution per site. Phylogenetic analyses were conducted in MEGA7 [49]. Trees made with neighbor-joining and maximum parsimony methods are very similar and can be seen in Supplementary Figure S4A,B.

For phylogenetic assessment of strain MS-P2^T^, the complete 16S rRNA gene sequence (1494 bp, NCBI acc. no. MZ358392) was retrieved from the sequenced whole genome. This sequence was 100% identical to the partial 16S rRNA gene sequence derived from an amplified PCR product (NCBI acc. no. MN600983) for this isolate, and the complete sequence was used in phylogenetic calculations. Pairwise nucleotide sequence similarity values for the 16S rRNA gene were calculated with the robust global sequence alignment algorithms in the EzTaxon server (https://www.ezbiocloud.net/ (accessed on 26 May 2021)) [48]. Phylogenetic trees were constructed with the MEGA 7 program [49] using the neighbor-joining, maximum parsimony, and maximum likelihood methods with Kimura’s two-parameter model [50] (Figure 5 and Supplementary Figure S4A,B). The maximum likelihood tree based on 16S rRNA gene sequences shows that strain MS-P2^T^ is a member of the genus *Elioraea* near the root of the *Acetobacteraceae* together with *E. tepidiphila*, *E. thermophila*, *E. rosea*, and *Elioraea* sp. strain “Yellowstone” (Figure 5). These five isolates form a monophyletic group that is an early-diverging clade within the family *Acetobacteraceae* (identity values of 90–92% for other members of this family). Similar results were obtained for phylogenetic trees calculated by the neighbor-joining and maximum parsimony methods (Supplementary Figure S4A,B). Surprisingly, strain MS-P2^T^ is closest in all cases to *E. rosea* with a pairwise sequence identity value of 97.99%, which is below the threshold value for novel species demarcation (proposed 16S rRNA gene sequence similarity threshold value of <98.6% [51]). Sequence identities to *E. tepidiphila* TU-7^T^ (=DSM 17972^T^), *E. thermophila* YIM 72297^T^, and *Elioraea* sp. strain “Yellowstone” were 97.26%, 96.58%, and 97.52%, respectively (Table 3). These findings support the proposal that strain MS-P2^T^ is a novel species within the genus *Elioraea.* Moreover, our findings support the proposal by Hördt et al. that the genus *Elioraea* should be placed in the family *Acetobacteraceae* [15,16]. 

**Figure 6:**
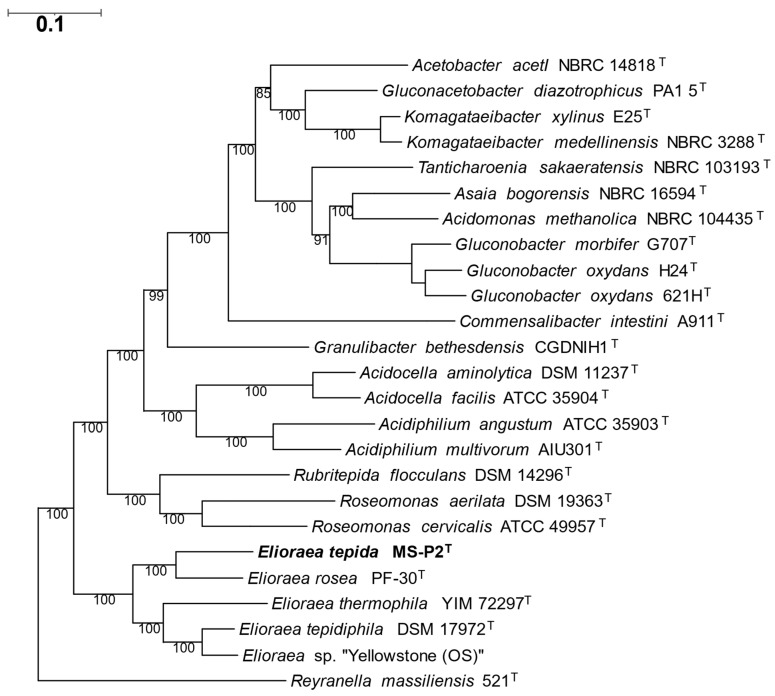
Inferred phylogenetic tree based on concatenated proteins using a set of 49 core universally conserved proteins/genes that shows the phylogenetic position of *Elioraea tepida* MS-P2^T^ within the genus *Elioraea* in comparison to selected members of the family *Acetobacteraceae*. The outgroup was *Rayranella massiliensis*, a member of the order *Hyphomicrobiales*. The other strains belonged to the order *Rhodospirillales* and the family *Acetobacteraceae*. The scale bar represents 0.10 substitution per site.

To assess the relationships among the five *Elioraea* strains currently available further and to assess the relationship of the genus *Elioraea* to other members of the *Alphaproteobacteria*, a phylogenetic analysis was performed that is based upon an alignment of a concatenation of 49 universally conserved proteins (Figure 6). As found in the ANI analysis described above, strain MS-P2^T^ was most closely related to the mesophilic strain *E. rosea*; and *E. tepidiphila* and *Elioraea* sp. “Yellowstone” were the second pair of more closely related strains. The five *Elioraea* spp. strains collectively form a monophyletic clade near the base of the strains forming the family *Acetobacteraceae* (Figure 6). 

It is interesting and surprising that strain MS-P2^T^ isolated from Mushroom Spring is distinctly different from *Elioraea* sp. “Yellowstone” isolated from nearby Octopus Spring. These two hot springs are separated by only about a quarter of a mile in the Lower Geyser Basin of Yellowstone National Park, and they are chemically very similar and have associated mat communities that are also quite similar [42]. However, *Elioraea* sp. “Yellowstone” from Octopus Spring is more similar to *E. tepidiphila* and not to *E. tepida* or *E. rosea*. 

Phenotypic, fatty acid, and genotypic characteristics of strain MS-P2^T^ and the three validly described species (*E. tepidiphila*, *E. thermophila*, *E. rosea*) [13,14,19] are summarized in Table 1, Table 2 and Table 3, respectively. These organisms generally share several common phenotypic traits, such as cell shape, swimming motility (except *E. thermophila*), moderate thermophily (except *E. rosea*), use of ubiquinone-10 as the respiratory quinone, production of catalase (except *E. rosea*, but *katG* is present in its genome), and the presence of cytochrome *c* oxidase. Similarities in cellular fatty acid and polar lipid composition also suggest that these organisms are closely related (Table 2). However, there are also important differences among these four isolates as follows: (1) The moderately thermophilic species were isolated from circum-neutral to slightly alkaline hot spring microbial mats, while the mesophilic *E. rosea* strain was isolated from floodwaters from a paddy field in South Korea. (2) *E. thermophila* is unique among the strains in having both phosphoribulokinase and type-1 ribulose 1,5-bisphosphate carboxylase-oxygenase, suggesting that this strain fixes CO_2_ by the Calvin–Benson–Bassham cycle. This provides an explanation for the unique ability among *Elioraea* spp. of *E. thermophila* to grow photoautotrophically [14]. (3) All strains including MS-P2^T^ produced BChl *a*, carotenoids, and a similar photosynthetic apparatus, although both *E. tepidiphila* and *Elioraea* sp. strain “Yellowstone” were initially reported to be non-pigmented [13,14,19]. (4) Like *E. rosea* and *E. tepidiphilia*, strain MS-P2^T^ showed optimal growth between pH 7.0–7.5, but *E. tepidiphila* prefers slightly more alkaline conditions (pH 8.0–8.5). (5) Strain MS-P2^T^ lacked C17:0, C16:0 2-OH, C18:0 2-OH, and 19:0 cyclo *ω*8 (Δ11:12) cellular fatty acids that were detected in other *Elioraea* spp. and uniquely possesses a significant amount of C15:0 *iso*, a fatty acid not found in any other *Elioraea* spp. (Table 2) (6) Strain MS-P2^T^ oxidizes a wide variety of sugars (e.g., fructose, fucose, galactose, mannose, melibiose, and psicose), while in general other *Elioraea* spp. cannot. (7) Strain MS-P2^T^ is the only *Elioraea* spp. strain that can hydrolyze gelatin. (8) Strain MS-P2^T^ could only oxidize proline, but *E. tepidiphila* can oxidize glutamate and glutamine as well as proline. Based on these phenotypic and genotypic differences and others (Table 1, Table 2 and Table 3), in addition to the phylogenetic and genomic differences among the strains described above, we propose strain MS-P2^T^ to be a new species belonging to the genus *Elioraea* with the suggested name *Elioraea tepida* (See description in Section 3.5). 

### 3.4. Distribution and Ecological Considerations

Ward et al. [52] reported a 16S rRNA sequence, designated “Type O”, in the microbial mats associated with Octopus Spring, Yellowstone National Park, WY, USA, in 1992. However, because the type species of the genus *Elioraea* was not described until 2008 [13], the Type O sequence (*Elioraea* spp.) initially could not be attributed to any specific organism. Later, using 16S rRNA amplicon sequencing and metagenomic sequencing of the upper green euphotic layer, a survey study of the mats of nearby Mushroom Spring provided the first evidence that members of the genus *Elioraea* were present in these hot-spring mat communities [17,53]. The 16S rRNA amplicon, which at that time was most similar to the 16S rRNA sequence of *E. tepidiphila*, could also be associated with a bin of sequences derived from the metagenome, which represented about 50% of the genome of the organism [17,53]. From the partial genomic information included in the *Elioraea* bin (Bin 22, OTU-46), it was apparent that the organisms associated with these sequences would likely have the capacity to synthesize BChl *a* and to produce bacterial reaction centers [17,18,53]. Strain MS-P2^T^, described herein, and strain “Yellowstone” [20] were subsequently isolated. Interestingly, the latter strain was reported to be non-pigmented [20], as was initially the case for *E. tepidiphila* [13], although the genomes of both suggested that they should produce BChl *a* and carotenoids like other *Elioraea* sp. strains [14,19]; this study. It is possible that these two closely related strains may only synthesize BChl *a* under specific growth conditions.

The physiology of *E. tepida* strain MS-P2^T^ described here and the distribution data from the 16S rRNA amplicon sequencing and metagenomic analyses do not necessarily agree [17,53]. The isolate clearly requires oxygen for growth and does not exhibit sensitivity to oxygen that might suggest it to be a microaerophile. However, 16S rRNA amplicon sequencing suggests that members of the genus *Elioraea* are not present or are rare in the uppermost green layer of the mat, where members of *Synechococcus* (*Cyanobacteria*) and *Roseiflexus* (*Chloroflexota*) spp. are the predominant organisms [7,17,18]. Instead, amplicon sequencing showed that sequences from *Elioraea* spp. are found in the undermat [17,53]. This observation indicates that strain MS-P2^T^ probably occurs naturally near but below the surface of the mat, i.e., in the upper portion of the undermat that also includes microaerophiles like *Chloracidobacterium thermophilum*. As previously noted, the genome encodes cytochrome *bd*- and *bb*_3_-type terminal oxidases, which characteristically have a higher affinity for oxygen than *aa*_3_-type oxidases. The ability of *E. tepida* to oxidize thiosulfate might also help to explain its location below the surface of the mats, away from the highest concentrations of oxygen. Sulfate reduction occurs away from the surface of the mat deeper in the anoxic zone and mostly at night [54]. Because *Elioraea* spp. are found in similar mat communities of both Mushroom and Octopus Springs, and because the two strains are similar but not very closely related, it is likely that these two strains play a specific role in the mat ecophysiology. However, at this time it is unclear what that role might be, and it is likewise uncertain whether *Elioraea* spp. can form specific associations with other microbes in these mat communities. Given the overall complexity of the chlorophototrophs in the Mushroom Spring mats, with at least 18 types of phototrophs identified to date [7,18], as well as dozens of ecotypes of the major mat inhabitants [55], deciphering the interactions among this panoply of phototrophs and the major chemoheterotrophs in the mat community will likely keep microbiologists busy for many years. 

### 3.5. Description of Elioraea tepida sp. nov.

*Elioraea*, type genus of the family *Elioraeaceae*; te.pi’.da; L. fem. adj. *tepida*, warm.

Cells are motile curved rods, 0.5 to 1.0 μm wide and 1.0 to 1.5 μm long, containing bacteriochlorophyll *a* and carotenoids of the spirilloxanthin series with spirilloxanthin predominant. Chemoheterotrophic growth occurred under strictly aerobic conditions, and light stimulated growth yield slightly; photoautotrophic growth with light, thiosulfate, and bicarbonate did not occur under oxic or anoxic conditions. Colonies in CTM medium containing succinate, acetate, and yeast extract are pinkish in color. Moderately thermophilic; the optimum growth temperature is about 45–50 °C and growth does not occur below 35 or above 55 °C. The optimum pH is between 7.0 and 7.5; growth does not occur at pH 5.5 or below nor above pH 10.5. Growth with nitrate by anaerobic respiration is not observed. Major fatty acids are 15:0 *iso* (7.8%), 16:0 (12.3%), 18:0 (30.0%), 18:1 *ω*7c (9.9%), 19:0 cyclo *ω*8c (13.0%), 18:1 *ω*7c 11-methyl (22.0%), and 18:0 3-OH (4.7%). Growth is stimulated by succinate and acetate (1.0 g L^−1^), and the following substrates can be oxidized in the presence of 0.1 g L^−1^ yeast extract: 3-methyl-d-glucose, citric acid, d-arabitol, d-fructose, d-fucose, d-galactose, d-mannose, d-melibiose, d-psicose, d-sorbitol, glucuronamide, hydroxybutyric acid, l-arabinose, l-proline, methyl pyruvate, *p*-hydroxy-phenylacetic acid, Tween 40, α-d-glucose, α-d-glucose-1-phosphate, β-hydroxy-d,l-butyric acid, and β-methyl-d-glucoside. Gelatin, aesculin, and pectin are hydrolyzed. Sensitive to troleandomycin, rifamycin SV, minocycline, lincomycin, vancomycin, nalidixic acid, and aztreonam in Biolog GEN III. Alkaline phosphatase, esterase (C4), esterase lipase (C8), leucine arylamidase, valine arylamidase, acid phosphatase, and naphthol-AS-BI-phosphohydrolase are produced. The mol % G + C content of the DNA is 70.6 mol %.

The type-strain, MS-P2^T^ (= JCM33060^T^ = ATCC TSD-174^T^), was isolated from Mushroom Spring in the Lower Geyser Basin of Yellowstone National Park, WY, USA (GPS coordinates: Lat.: 44.5387, Long.: −110.798).

## Figures and Tables

**Table 1:** Differential characteristics of species in the genus *Elioraea*. Positive [+], negative [−], not determined [ND]. All species can synthesize bacteriochlorophyll *a* and carotenoids, are pink in color, are positive for cytochrome *c* oxidase, and employ ubiquinone-10 as the major respiratory quinone.

Property	^1^*E. tepidiphila* DSM17972^T^	^2^*E. thermophila* YIM 72297^T^	^3^*E. rosea* PF-30A^T^	*E. tepida* MS-P2^T^
Cell morphology	Rods	Curved rods	Rods	Curved rods
Cell size (width × length (µm))	0.5–1.0 × 1.0–1.5	0.7–0.9 × 2.2–3.2	1.0–1.2 × 2.2–2.6	0.5–1.0 × 1.0–1.5
Motility (flagella genes present)	+	−	+	+
Temperature optimum (range) °C	45–50 (30–50)	55 (45–60)	28 (20–40)	45–50 (>35–<55)
pH optimum (range)	8.0–8.5 (6.0–9.8)	7.0–7.5 (5.0–9.0)	7.0 (5.0–9.0)	7.0–7.5 (6.0–10.0)
Catalase	+	+	–(but *katG* is present) ^4^	+
Photoautotrophic growth	−	+	−	−
Substrate Utilization ^5^				
D-Fructose	−	+	−	+
D-Fucose	−	−	−	+
D-Galactose	−	−	−	+
D-Mannose	−	−	−	+
D-Melibiose	−	−	−	+
D-Psicose	−	−	ND	+
Glutamate	+	ND	ND	−
Glutamine	+	ND	ND	−
Proline	+	ND	ND	+
Compounds Hydrolyzed				
Starch	+	−	−	−
Gelatin	−	−	−	+
Aesculin	−	−	+	+
Pectin	−	ND	ND	+
Casein	−	−	−	ND

^1^ Data are from references [13,14]. ^2^ Data are from reference [14]. ^3^ Data are from reference [19]. ^4^ *katG*, catalase. ^5^ Positive reactions detected in Biolog Gen III plates for strain MS-P2^T^ reflect a chemical transformation and can be due to enhanced respiration and/or growth and do not necessarily mean that a substrate supports growth. The complete list of compounds tested is available online at https://www.biolog.com/wp-content/uploads/2020/04/00P_185_GEN_III_MicroPlate_IFU.pdf (accessed on 21 December 2021).

**Table 2:** Fatty acid composition of *Elioraea* species type strains.

Fatty Acid	^1^*E. tepidiphila* DSM17972^T^	^2^*E. thermophila* YIM 72297^T^	^3^*E. rosea* PF-30A^T^	*E. tepida* MS-P2^T^
14:0	0.3%	–	–	–
15:0 *iso*	–	–	–	7.8%
16:0	5.4%	12.6%	3.6%	12.3%
17:0	0.4%	–	1.6%	–
16:0 2-OH	1.9%	5.4%	2.7%	–
18:1 *ω*7c	19.0% ^4^	30.1%	35.7%	9.9%
18:0	24.8%	35.8%	22.7%	30.0%
18:1 *ω*7c 11-methyl	8.0%	–	3.4%	22.0%
19:0 cyclo *ω*8c	12.4%	4.2%	5.7%	13.0%
18:0 2-OH	0.6%	1.5%	2.7%	–
18:0 3-OH	3.8%	2.5%	2.9%	4.7%
19:0 2-OH cyclo *ω*8 (Δ11:12)	18.6%	–	7.9%	–

^1^ Data are from references [13,14]. ^2^ Data are from reference [14]. ^3^ Data are from reference [19]. ^4^ The 18:1 *ω*7c value for *C. tepidiphila* may also include 18:1 *ω*6c, which could not be resolved.

**Table 3:** Selected genomic characteristics of strains and species in the genus *Elioraea*.

Genomic Properties	^1^*E. tepidiphila* DSM17972^T^	^2^*E. thermophila* YIM 72297^T^	^3^*E. rosea* PF-30A^T^	^4^*Elioraea* sp. “Yellowstone”	*E. tepida* MS-P2^T^
Size (bp)	>4,304,240	3,029,970	>4,487,660	>3,824,070	3,367,643
Proteins (CDS)	>4014	2845	>4189	>3647	3083
rRNA genes	3	3	3	3	3
tRNAs	48	46	46	46	46
DNA Mol % G + C	71.3	70.9	69.9	72.4	70.6
Average nucleotide identity (%) ^5^	75.06	73.34	78.59	75.84	––
Digital DNA-DNA hybridization (%) ^5^	19.8	18.7	21.9	20.5	––
16S rRNA % identity ^6^	97.26	96.58	97.99	97.52	––
Nitrate reductase (NarG) /growth with nitrate	+/−	−/−	+/−	+/ND	−/−
RubisCO (RbcLS) and Phosphoribulokinase (PRK)	−	+	−	−	−
Urease (UreABCDEFGJ)	−	+	+	−	+
Thiosulfate oxidation (SoxBCDYZ)	+	+	−	+	+

^1^ Data are from references [13,14], and GenBank entry NZ_ARKI00000000. ^2^ Data are from reference [14]. ^3^ Data are from reference [19]. ^4^ Data are from reference [20]. ^5^ Values are the results from comparisons with the *E. tepida* MS-P2^T^ genome. ^6^ Values are the results from comparisons with the *E. tepida* MS-P2^T^ 16S rRNA gene sequence.

## Data Availability

The GenBank/EMBL/DDBJ accession numbers for the 16S rRNA gene sequences of strain MS-P2^T^ are MN600983 and MZ358392. The genome sequence data for the type-strain are available under the GenBank/EMBL/DDBJ accession number CP076448. *Elioraea tepida* strain MS-P2^T^ has been deposited in the Japan Collection of Microorganisms (=JCM 33060^T^) and in the American Type Culture Collection (=ATCC TSD-174^T^).

## Supplementary Materials

The following are available online at https://www.mdpi.com/article/10.3390/microorganisms10010080/s1, Figure S1 Appearance of *Elioraea tepida* strain MS-P2^T^. Figure S2 HPLC analysis of pigments extracted from *Elioraea tepida* strain MS-P2^T^. Figure S3 Thin-layer chromatogram of polar lipids of *Elioraea tepida* strain MS-P2^T^. Figure S4 Neighbor-joining and maximum parsimony phylogenetic trees based upon 16S rRNA sequences. Table S1 Fatty acid composition of Elioraea species type strains.
