# Supplementary material for: Elioraea tepida, sp. nov., a Moderately Thermophilic Aerobic Anoxygenic Phototrophic Bacterium Isolated from the Mat Community of an Alkaline Siliceous Hot Spring in Yellowstone National Park, WY, USA
Source: Microorganisms. 2021 Dec 31;10(1):80. doi: 10.3390/microorganisms10010080 (PMC8781829; doi:10.3390/microorganisms10010080)
Supplement: Supplementary file 1 [file microorganisms-10-00080-s001.zip › microorganisms-1517308-supplementary.pdf]

Supplementary Material: Figures S1 to S4

***Microorganisms* MDPI**

Elioraea tepida, sp. nov., a moderately thermophilic aerobic anoxygenic phototrophic bacterium isolated from the mat community of an alkaline siliceous hot spring in Yellowstone National Park, WY, USA

Mohit Kumar Saini[1#], Shohei Yoshida[1#], Aswathy Sebastian[2], Eri Hara[3], Hideyuki Tamaki[3], Nathan T. Soulier[4], Istvan Albert[2,4], Satoshi Hanada[1], Marcus Tank[1,4,5]*, and Donald A. Bryant[4]*

[1]*Department of Biological Sciences, Tokyo Metropolitan University, Hachioji, Tokyo, Japan;* [2]*The Huck Institutes for the Life Sciences, The Pennsylvania State University, University Park, PA United States;* [3]*Bioproduction Research Institute, National Institute of Advanced Industrial Science and Technology (AIST), 1–1–1, Higashi, Tsukuba, Ibaraki, 305–8566, Japan;* [4]*Department of Biochemistry and Molecular Biology, The Pennsylvania State University, PA, United States;* [5]DSMZ – German Culture Collection of Microorganisms and Cell Cultures, GmbH Inhoffenstraße 7B, Braunschweig, Germany

[#]Both authors equally contributed to the study

*Correspondence: Marcus Tank, mat19@dsmz.de, +49-531-2616-395; Donald A. Bryant, dab14@psu.edu, +1-814-777-9699

Supplementary Figure S1.

a

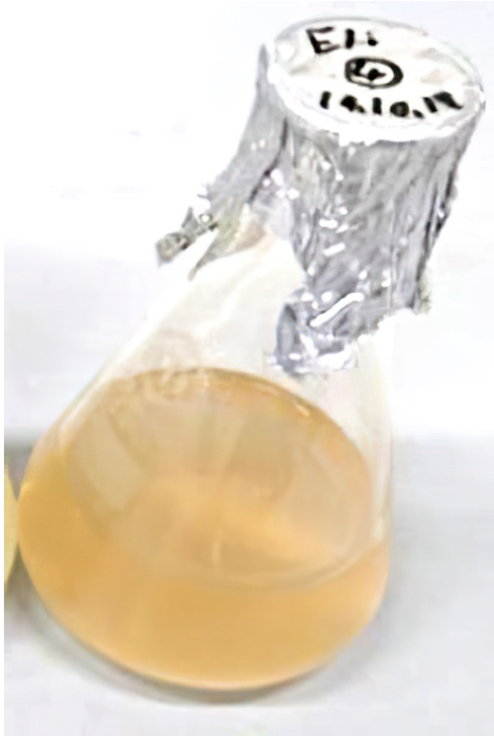

Axenic culture of
***Elioraea tepida* MS-P2^T**

b

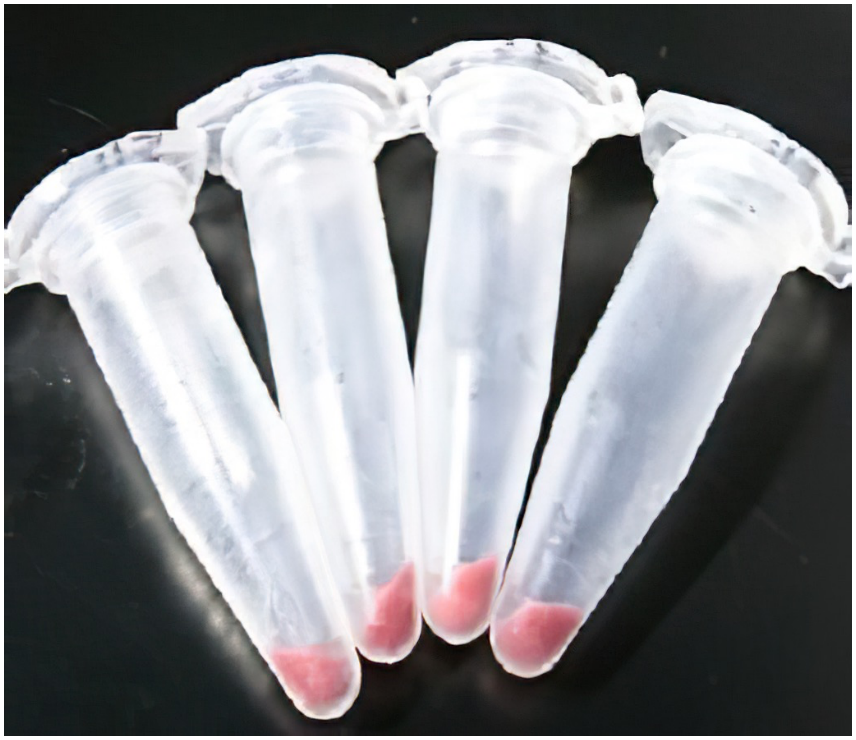

Pellets of *Elioraea tepida* strain MS-P2^T from a liquid culture

Supplementary Figure S1. Appearance of *Elioraea tepida* strain MS-P2^T. Appearance of *Elioraea tepida* strain MS-P2^T cells in liquid culture (**a**) and after centrifugation to form cell pellets (**b**). Cells exhibit the pinkish coloration due to the synthesis of BChl *a* and carotenoids.

Supplementary Figure S2.

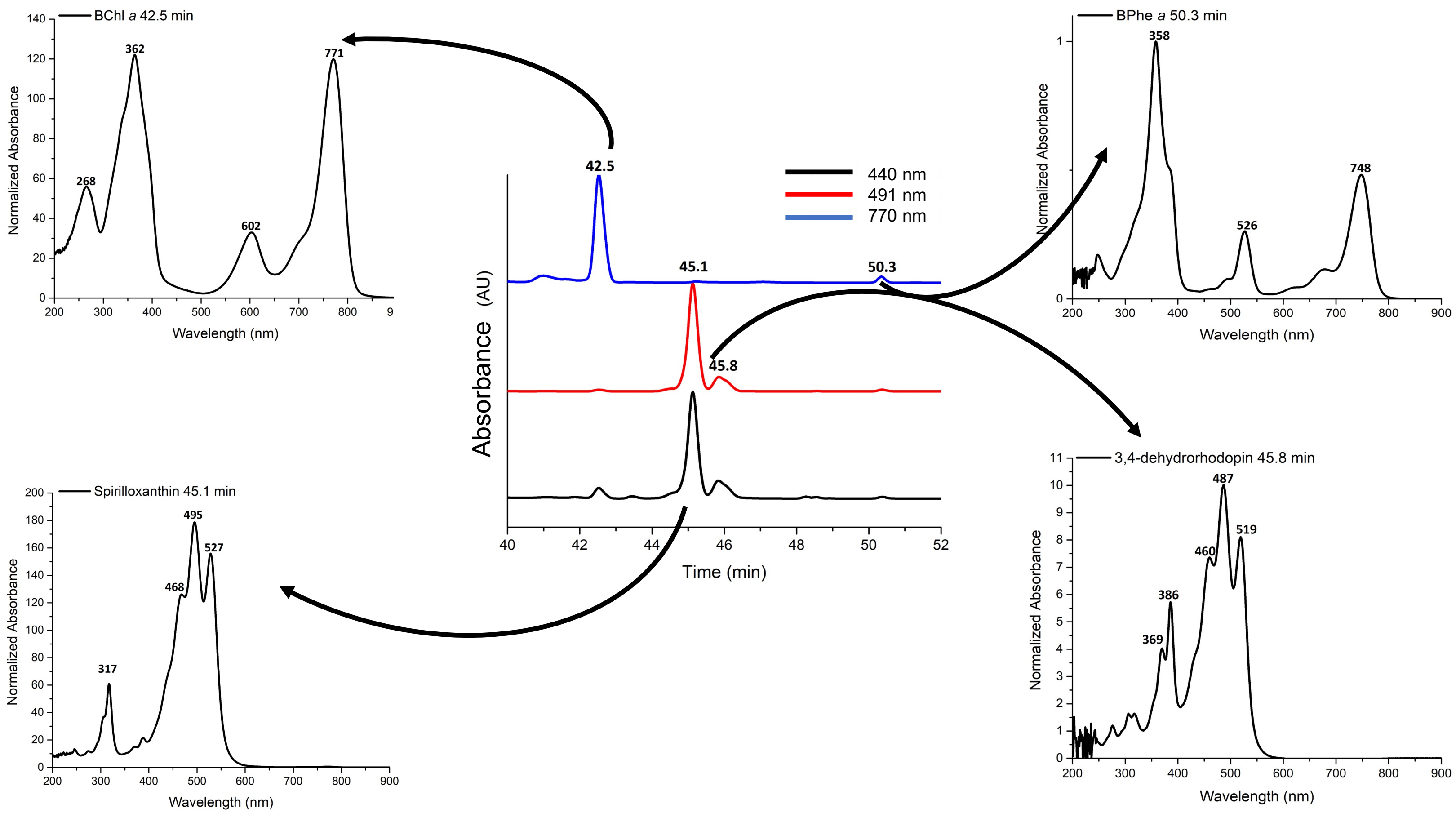

Supplementary Figure S2. HPLC analysis of pigments extracted from *Elioraea tepida* strain MS-P2^{T}. The central panel shows a portion of the elution profile monitored at 770 nm (blue line), 491 nm (red light) and 440 nm (black line). The absorbance spectra of selected peaks are shown at the periphery (arrows). BChl *a* elutes at 42.5 min, BPheo *a* elutes at 50.3 min; spirilloxanthin elutes at 45.1 min; and 3,4-dehydrorhodopin elutes at 45. 8 min.

Supplementary Figure S3.

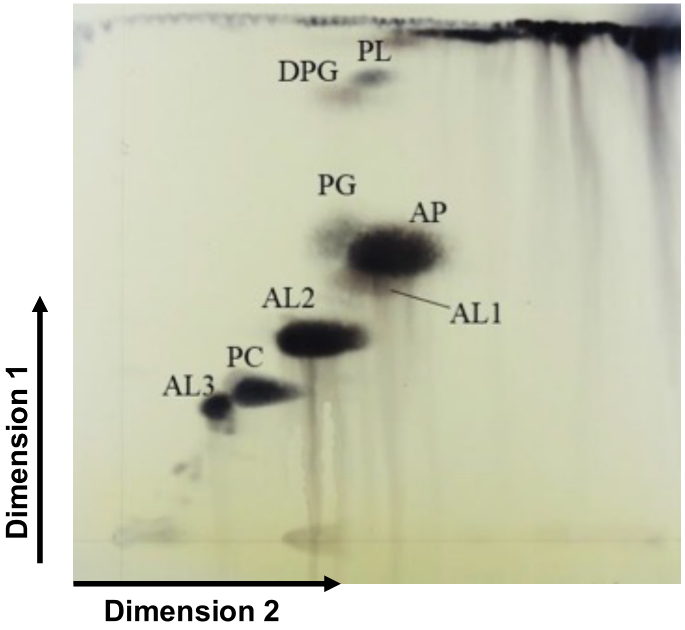

Two-dimensional thin-layer chromatogram of the total polar lipids of strain MS-P2^T.

PL: unidentified phospholipid
DPG: diphosphatidylglycerol
PG: phosphatidylglycerol
AP: unidentified aminophospholipid (possibly phosphatidylethanolamine)
PC: phosphatidylcholine
AL1, AL2, and AL3: unidentified amino lipids 1, 2, and 3

Supplementary Figure S3. Thin layer chromatogram of analyzing polar lipids of *Elioraea tepida* strain MS-P2^T. The identified lipids are indicated at the right.

Supplementary Figure S4

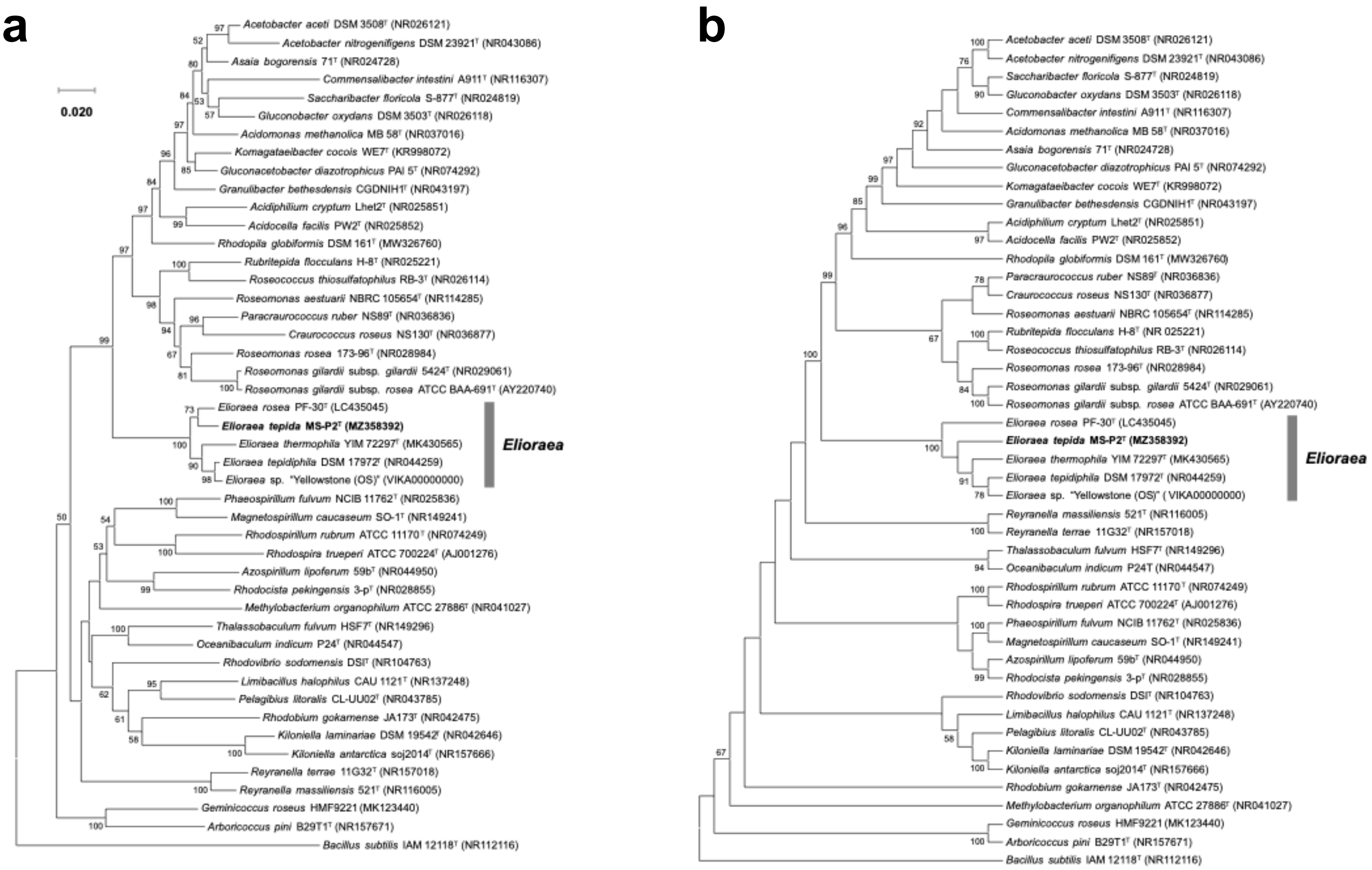

Supplementary Figure S4. Phylogenetic trees based upon 16S rRNA sequences.

Phylogenetic trees based on 16S rRNA gene sequences constructed by the neighbor-joining (NJ) method (**Figure S4a**) and maximum parsimony (MP) (**Figure S4b**) based on the Kimura 2-parameter model [48] showing the phylogenetic position of strain MS-P2^T within the order *Rhodospirillales*. Robustness of the trees was tested by bootstrapping (1000 and 100 resamplings for NJ and MP, respectively, values >50 are given at the nodes). *Bacillus subtilis* IAM 12118^T is used as outgroup. The scale bar represents 0.02 substitutions per site. Phylogenetic analyses were conducted in MEGA7 [47].

Supplementary Table S1.

Supplemental Table S1. Fatty acid composition of *Elioraea* species type strains.

Fatty Acid	[1]*E. tepidiphila* DSM17972^T	[2]*E. thermophila* YIM 72297^T	[3]*E. rosea* PF-30A^T	*E. tepida* MS-P2^T
14:0	0.3%	–	–	–
15:0 *iso*	–	–	–	7.8%
16:0	5.4%	12.6%	3.6%	12.3%
17:0	0.4%	–	1.6%	–
16:0 2-OH	1.9%	5.4%	2.7%	–
18:1 *ω*7c	19.0%[4]	30.1%	35.7%	9.9%
18:0	24.8%	35.8%	22.7%	30.0%
18:1 *ω*7c 11-methyl	8.0%	–	3.4%	22.0%
19:0 cyclo *ω*8c	12.4%	4.2%	5.7%	13.0%
18:0 2-OH	0.6%	1.5%	2.7%	–
18:0 3-OH	3.8%	2.5%	2.9%	4.7%
19:0 2-OH cyclo *ω*8 (Δ11:12)	18.6%	–	7.9%	–

[1]Data are from references [12] and [13].
[2]Data are from reference [13].
[3]Data are from reference [18].
[4]The 18:1 *ω*7c value for *E. tepidiphila* may also include 18:1 *ω*6c, which could not be resolved.
